# Mechanistic Study of Copper-Catalyzed C-H Hydroxylation/C-S Coupling by ESI-HR MS and DFT Calculations

**DOI:** 10.3390/molecules22111912

**Published:** 2017-11-06

**Authors:** Runsheng Xu, Rongrong Cai, Sixian Zhou, Zhuoda Zhou, Beibei Li, Dihui Xu

**Affiliations:** Department of Biology and Environment, Jiyang College of Zhejiang A&F University, Shaoxing 311800, China; cairongrong@163.com (R.C.); sixianzhou@126.com (S.Z.); zhouzhuoda@163.com (Z.Z.); libeibei20172017@163.com (B.L.); xudihui2017@sina.com (D.X.)

**Keywords:** reaction mechanism, Cu-catalyzed, C-H hydroxylation/C-S coupling, ESI-HR MS, DFT

## Abstract

The reaction mechanism of Cu-catalyzed C-H hydroxylation/C-S coupling was studied using electrospray ionization high resolution mass spectrometry (ESI-HR MS) and density functional theory calculations (DFT). Notably, a series of Cu^I^ and Cu^III^ complexes were observed as key intermediates and identified using ESI-HR MS. Furthermore, a catalyst cycle involving proton abstraction/oxidative addition/reductive elimination was proposed. This study is important and valuable with respect to C-H functionalization.

## 1. Introduction

C-H bond activation is a hot topic in modern organic chemistry [1,2,3]. There are many methods to activate the C-H bonds of molecules, although C-H activation in pure hydrocarbons is still a challenge [4,5,6]. Most methods involve transition-metal catalysis, where a continuum of reaction mechanisms exists. Some C-H activation reactions have been shown to proceed much more efficiently and selectively through ligand direction [7,8,9]. Such assistance is well known for metal catalysts with ligands [10]. One of the first examples of such a reaction was reported in 1955 by Winstein and Traylor for the acetolysis of diphenylmercury in acetic acid [11]. The investigation of C-H bond activation mechanisms can provide information on key reaction factors, allowing for the design of new reactions [12,13,14]. However, C-H bond activation mechanisms are difficult to study due to the complex interactions between the reaction parameters [15,16]. Early mechanistic studies proposed an electrophilic aromatic substitution mechanism [17,18,19], for which large primary kinetic isotope effects were observed [20].

**Scheme 1:**
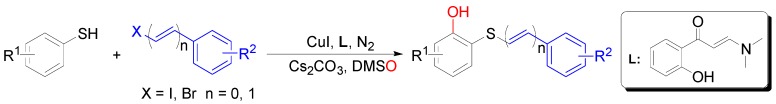
Cu-catalyzed C-H hydroxylation/C-S coupling.

The application of electrospray ionization high resolution mass spectrometry (ESI-HR MS) provides key intermediates with mass information. Density functional theory (DFT) can compute the reaction pathways with respect to different energy profiles for intermediate species and transition states [21,22]. We reported on the highly efficient Cu-catalyzed C-H hydroxylation/C-S coupling (Scheme 1) [23,24]. The broad substrate scope coupled with the mild reaction conditions prompted us to further investigate the reaction mechanism. To this end, we employed a number of different strategies including an isotope labeling study, mass spectrometry experiments, and DFT calculations. Based on ESI-HR MS experiments and DFT calculations, we propose a proton abstraction/oxidative addition/reductive elimination reaction mechanism of Cu-catalyzed C-H hydroxylation/C-S coupling.

## 2. Results and Discussion

**Scheme 2:**
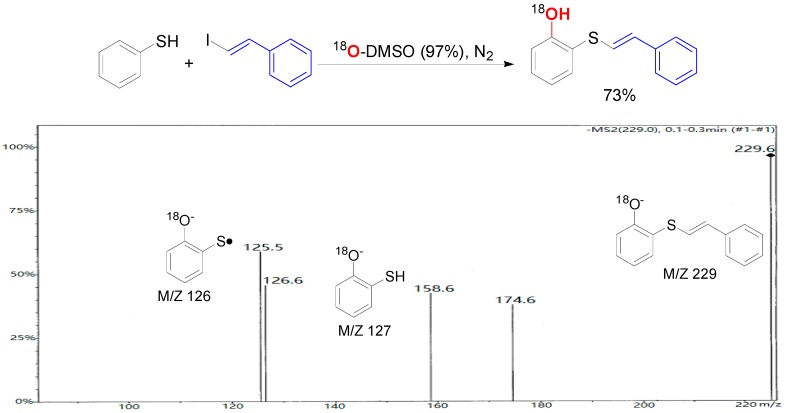
Isotope labeling study.

The reaction of vinyl halides with thiophenol was chosen as the model reaction (Scheme 1, *n* = 1). The solution dimethyl sulfoxide was identified as a critical component in C-H hydroxylation. Based on this, alabeling study was employed to investigate the possible oxygen source of the hydroxy moiety (Scheme 2). The product was inspected by ESI-MS/MS after the completion of the reaction. The results showed that the oxygen source was dimethyl sulfoxide.

**Scheme 3:**
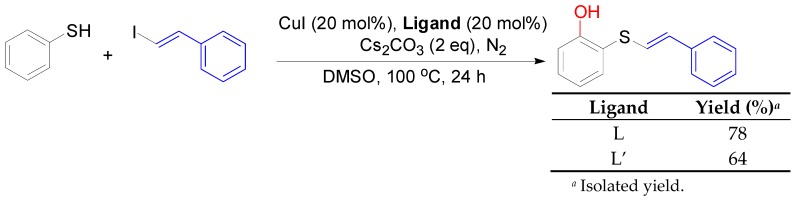

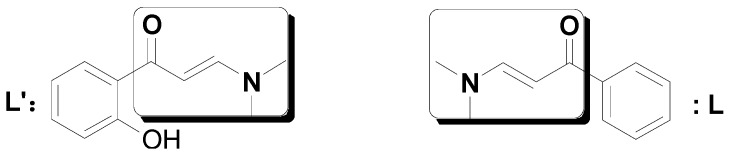
Identification of the catalyzing segment.

In order to investigate which segment acted as the catalyst, the thiophenol and 1-((*E*)-2-iodovinyl)benzene reaction was catalyzed by the enaminone ligands **L** and **L’** (Scheme 3). Ligand A was used in the reactions which we previously reported [23,24]. They gave medium yields, indicating that the hydroxyl group may not play a role in this reaction.

**Scheme 4:**
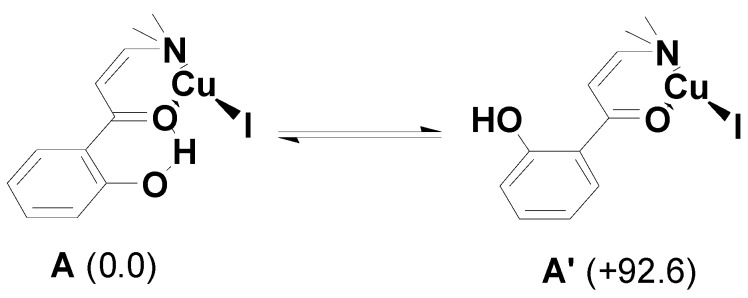
**LCuI** complex formation. Values in parentheses are free energies in kJ/mol.

**Figure 1:**
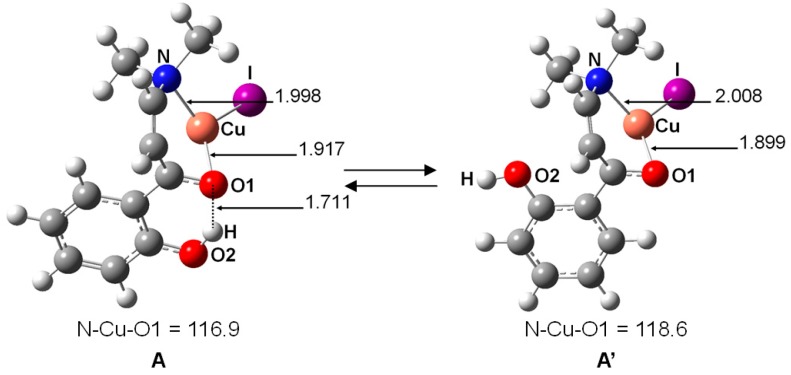
Optimized structures for **LCuI** complexes (bond lengths in Å).

Subsequently, we used electrospray ionization high resolution mass spectrometry (ESI-HRMS) to monitor the reaction. The peak at 254.0230 (*m/z*, [**A** − I]^+^, please see Supplementary Materials Figure S3) corresponding to the coordination of **L** to CuI gave sufficient evidence for the formation of a catalyst (For more details see, Supporting Information) [25,26,27]. In addition, the DFT calculation also confirmed two possible isomeric species in the reaction mixture (Scheme 4). The isomer bearing an intramolecular hydrogen bond was named intermediate **A**, and another was named intermediate **A’** (Figure 1). The DFT calculations indicated that the formation of intermediate **A** (0.0 kJ/mol) was more favorable than the formation of intermediate **A’** (92.6 kJ/mol). The intermediate **A** was considered as the intermediate for the nucleophile coordination step. Thus, the hydroxyl group in **L** is likely to enhance the ligand coordination ability.

**Scheme 5:**
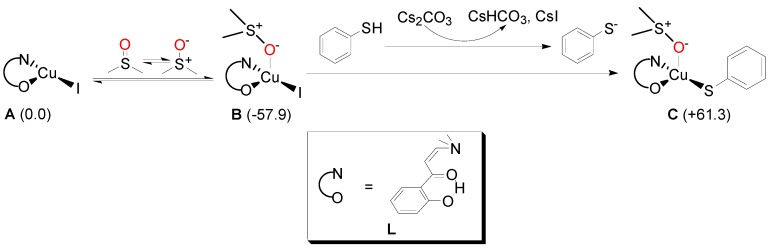
Nucleophile coordination and ligand exchange.

**Figure 2:**
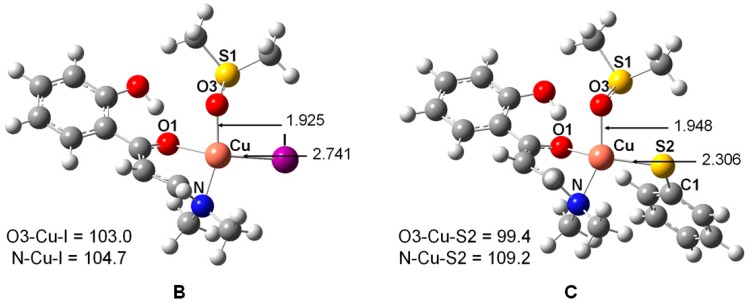
Optimized structures for intermediates **B** and **C**.

It has been reported that the S atom easily coordinates with the Cu (Scheme 5) [28,29,30]. The coordination of DMSO with intermediate **A** resulted in the formation of intermediate **B** ([**B** − I]^+^, *m/z* = 332.0387, please see Supplementary Materials Figure S4) [31,32]. It should be noted that in this step we only considered DMSO as a neutral nucleophile. The free energy change value was −57.9 kJ/mol, which indicatesthat the reaction is a favorable thermodynamic process. Cs_2_CO_3_ in DMSO readily deprotonates thiophenol to provide thiophenolate [33,34]. Consequently, ligand exchange occurred before the proton abstraction was identified by the DFT calculations (Figure 2). The structures of intermediates **B** and **C** ([**C** + H]^+^, *m*/*z* = 442.0557, please see Figure S5) were identified by DFT calculation (Figure 2).

**Scheme 6:**
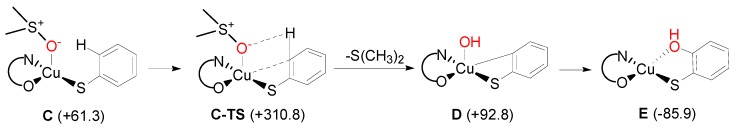
Proton abstraction and hydroxyl group transfer.

**Figure 3:**
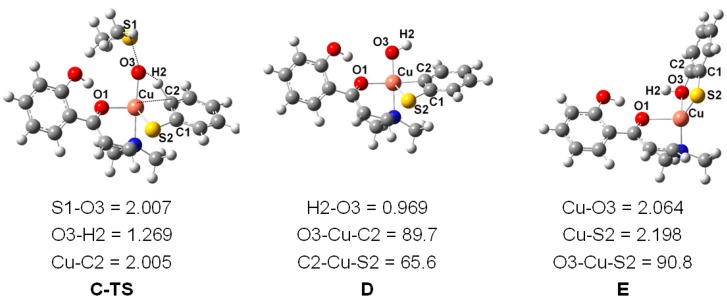
Optimized structures for **C-TS** and intermediates **D** and **E**.

The intermediate **C** reacted with aryl thiols, producing the five-coordinate CuI intermediate **D** ([**D** + Na]^+^, *m*/*z* = 402.0201, please see Figure S6) via proton abstraction (Scheme 6). Interestingly, the DFT calculations indicated that the transition state **C-TS** existed, and also showed that proton abstraction was a highly endothermic process. Under the influence of Cu, DMSO became very unstable (Figure 3). Then, dimethyl sulfide became automatically dissociated [35,36,37].The four-coordinate CuI intermediate **E** ([**E** + Na]^+^, *m/z* = 402.0201, please see Figure S7) was produced by the hydroxyl group transfer. The reaction barrier of 310.8 kJ/mol in the transition state was high. However, the reaction temperature was 100 °C in our reaction system, which was sufficient to provide the energy required for this reaction. On the other hand, the ligand coordination ensured that the reaction proceeded smoothly. This transformation was also thermodynamically favorable ((−85.9) − 92.8 = −178.8 kJ/mol).

**Scheme 7:**
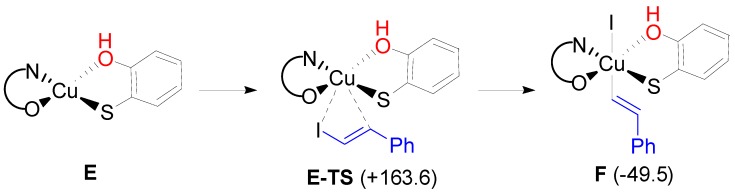
Oxidative addition.

**Figure 4:**
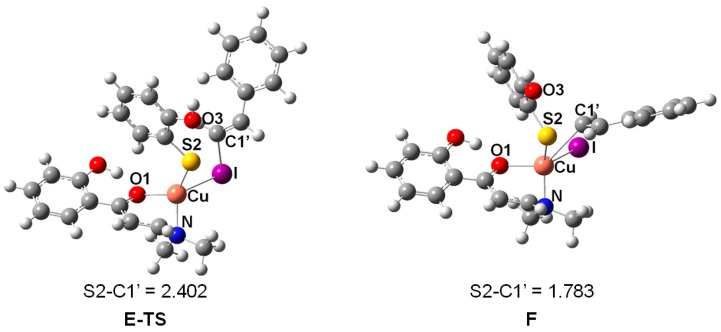
Optimized structures for intermediates **E-TS** and **F**.

Next, intermediate **E** reacted with vinyl halides by oxidative addition, producing the Cu^II^I intermediate **F** (Scheme 7, [**F** − I]^+^, *m/z* = 483.0937, please see Figure S8). The DFT calculations indicated that the transition state **F-TS** existed in the oxidative addition (Scheme 7) [38]. After that, the intermediate **F** underwent reductive elimination to obtain the product and intermediate **A**, which re-enters the catalytic cycle. Our calculations showed that the elimination step was largely thermodynamically favorable (Figure 4).

**Scheme 8:**
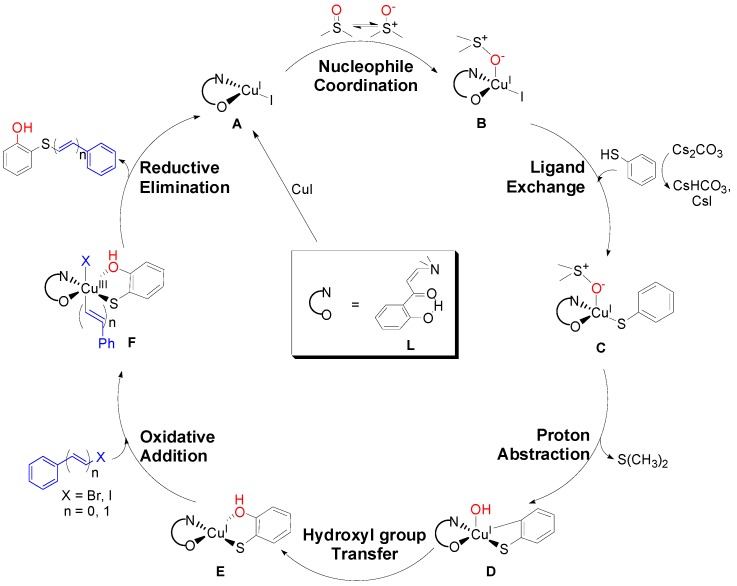
Proposed mechanism.

**Figure 5:**
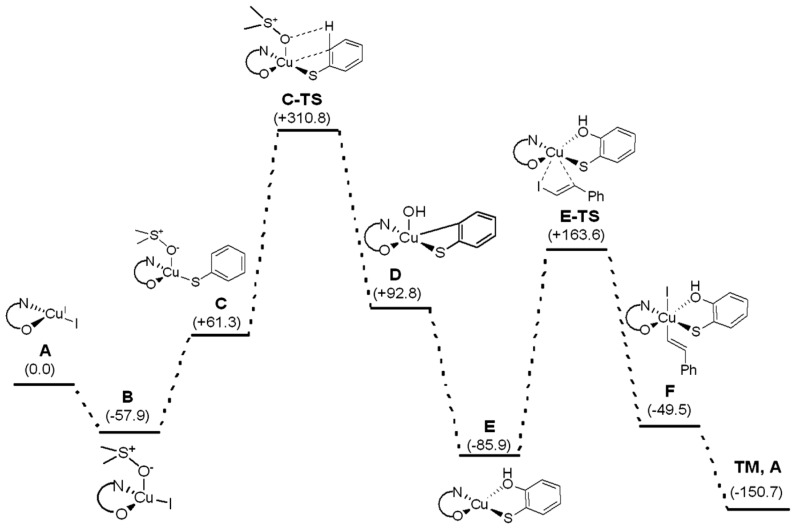
Energy profile of Cu-catalyzed direct C-H hydroxylation/C-S coupling.

Based on the above experimental and computational studies, an overall catalytic cycle is proposed in Scheme 8. After excluding the more unstable isomer **A’**, the coordination of the intermediate **A** with DMSO generated the reactive intermediate **B**. Next, intermediate **B** produced the intermediate **C** via the ligand exchange step, which was favorable in thermodynamics. Then, the five-coordinate intermediate **D** was formed by the proton abstraction step and the four-coordinate intermediate **E** was formed by the hydroxyl group transfer step. Finally, oxidative addition produced a Cu^III^ intermediate. After that, the intermediate **F** underwent reductive elimination to give the product, which was largely thermodynamically favorable. The concomitantly formed intermediate **A** re-entered the catalytic cycle. Energy profile of Cu-catalyzed direct C-H hydroxylation/C-S coupling, please see Figure 5.

## 3. Experimental Section

### 3.1. General Procedure for the Preparation of Ligand ***L***

Dimethylformamide dimethylacetal (DMFDMA) (1.19 g, 10 mmol) and 1-(2-hydroxyphenyl) ethanone (1.36 g, 10 mmol) were dissolved in *p*-xylene (2 mL). The mixture was refluxed over a period of 10 h, during which time the formation of a yellow precipitate occurred. The precipitate was filtered out and washed with petroleum ether three times. The solid was vacuum-dried, and 1.79 g (94% yield) of a yellow solid, **L** ((*E*)-3-(dimethylamino)-1-(2-hydroxyphenyl)prop-2-en-1-one), was obtained. ^1^H-NMR (500 MHz, DMSO-*d*_6_):δ14.51 (s, 1 H), 7.92–7.90 (t, *J* = 7.5 Hz, 2 H), 7.37–7.34 (t, *J* = 7.8 Hz, 1 H), 6.83 (d, *J* = 2.0 Hz, 2 H), 5.98–5.95 (d, *J* = 12 Hz, 1 H), 3.19 (s, 3 H), 2.98 (s, 3 H); ^13^C-NMR (125 MHz, DMSO-*d*_6_): δ 191.1, 163.6, 156.7, 134.9, 129.9, 121.2, 119.1, 118.7, 90.4, 46.1, 38.6.

### 3.2. General Procedure for the Preparation of 2-(Styrylthio)phenols

A mixture of thiophenol (55 mg, 0.5 mmol), 1-((*E*)-2-iodovinyl)benzene (138 mg, 0.6 mmol), CuI (19 mg, 20 mol %), **L** (19 mg, 20mol %), and Cs_2_CO_3_ (326 mg, 2 equivalent) in DMSO (4 mL) was stirred in N_2_ at 100 °C for 24 h. After completion of the reaction, the mixture was quenched with saturated salt water (10 mL), and the solution wa sextracted with ethyl acetate (3 × 10 mL). The organic layers were combined and dried over sodium sulfate. The pure product 2-(styrylthio)phenol (89 mg, 78% yield) was obtained by flash column chromatography on silica gel.

### 3.3. General Procedure for Computational Details

B3LYP density functional theory (DFT) calculations were performed using the Gaussian 03 program [39]. The effective core potential (ECP) basis sets were used for I, and 6-311G* basis sets were used for C, H, O, S and Cu. No symmetry constraint was imposed in the optimization. All reactants, intermediates, and products were identified as true minima by the absence of imaginary frequencies. Compounds had multiple conformations; efforts were made to find the lowest-energy conformation by comparing the structures optimized from different starting geometries. The transition state (TS) was identified by the presence of one single imaginary vibration frequency and the normal vibrational mode. In addition, transition states were confirmed by intrinsic reaction coordinate(IRC) calculations. Unscaled zero-point energies were included for different species. All the gas-phase free energies (kJ/mol) reported in this paper correspond to the reference state of **A** at 373 K. The optimized structures were shown by Gauss View (Version 3.09) software to give high-quality images of these structures.

## 4. Conclusions

The reaction mechanism of Cu-catalyzed C-H hydroxylation/C-S cross-couplingwas investigated using a combination of electrospray ionization mass spectrometry, density functional theory calculations, and an isotope labeling study. Notably, a series of Cu^I^ and Cu^III^ complexes as key intermediates were observed and identified by ESI-HR MS. Furthermore, the catalyst cycle involving proton abstraction/oxidative addition/reductive elimination was proposed. This study should provide valuable mechanistic insight to other Cu-catalyzed C-H functionalization mechanisms.

## Supplementary Materials

Supplementary Materials are available online.
