# Supplementary material for: Mechanistic Study of Copper-Catalyzed C-H Hydroxylation/C-S Coupling by ESI-HR MS and DFT Calculations
Source: Molecules. 2017 Nov 6;22(11):1912. doi: 10.3390/molecules22111912 (PMC6150210; doi:10.3390/molecules22111912)
Supplement: Supplementary file 1 [file molecules-22-01912-s001.pdf]

Supporting Information

Mechanistic Study of Copper-catalyzed C-H Hydroxylation/C-S Coupling by ESI-HR MS Spectrometry and DFT Calculations

Runsheng Xu*, Rongrong Cai, Sixian Zhou, Zhuoda Zhou, Beibei Li, Dihui Xu

Department of Biology and Environment, Jiyang College of Zhejiang A &F University, Shaoxing 311800, Zhejiang China

**E-mail: 20140041@zafu.edu.cn*

Contents

Experimental details.. S2

ESI-HR MS of the intermediates... S4

Geometries, total energies and zero point energy corrections................ S8

Experimental details

General information

All reagents used in the experiment were obtained from commercial sources and used without further purification. Unless otherwise noted, all reactions were carried out at N_2 atmosphere. Analytical thin layer chromatography (TLC) employed glass 0.25 mm silica gel plates. All NMR spectra were recorded on Bruker AVANCE DMX-500 spectrometry at 500 MHz and 125 MHz for ^{1}H and ^{13}C NMR in d^6-DMSO, respectively. The NMR chemical shift was reported in ppm relative to 2.50 and 40.70 ppm of DMSO as the standards of ^{1}H and ^{13}C NMR, respectively. The ^{1}H NMR spectra were reported in delta (δ) units, parts per million (ppm) downfield from the internal standard. Coupling constants are reported in Hertz (Hz). Mass spectras were performed on a Bruker Esquire 3000plus mass spectrometer equipped with ESI interface and ion trap analyzer. The ESI-HR MS were tested on Bruker 7-tesla FT-ICR MS equipped with an electrospray source.

General procedure for preparation of ligand L and ligand L′

Dimethylformamide dimethylacetal (DMFDMA) (1.19 g, 10 mmol) and 1-(2-hydroxyphenyl) ethanone (1.36 g, 10 mmol) were dissolved in *p*-xylene (2 mL). And the mixture was refluxed during a period of 10 hours, during which time the formation of yellow precipitate. The precipitate was filtered out and washed with petroleum ether three times. The solid was vacuum-dried, and 1.79 g (94% yield) of a yellow solid **L** ((*E*)-3-(dimethylamino)-1-(2-hydroxyphenyl)prop-2-en-1-one) was obtained. ^{1}H NMR (500 MHz, d^6-DMSO): δ 14.51 (s, 1 H), 7.92-7.90 (t, *J* = 7.5 Hz, 2 H), 7.37-7.34 (t, *J* = 7.8 Hz, 1 H), 6.83 (d, *J* = 2.0 Hz, 2 H), 5.98-5.95 (d, *J* = 12 Hz, 1 H), 3.19 (s, 3 H), 2.98 (s, 3 H); ^{13}C NMR (125

MHz, d^6-DMSO): δ 191.1, 163.6, 156.7, 134.9, 129.9, 121.2, 119.1, 118.7, 90.4, 46.1, 38.6.

General procedure for computational details

B3LYP Density Functional Theory (DFT) calculations were preformed on the Gaussian 03 program.[6] The Effective Core Potential (ECP) basis sets were used for I, and 6-311G* basis sets for C, H, O, S and Cu. No symmetry constraint was imposed in the optimization. All reactants, intermediates and products were identified as true minima by the absence of imaginary frequencies. Compounds had multiple conformations, efforts were made to find the lowest-energy conformation by comparing the structures optimized from different starting geometries. Transition State (TS) was identified by the presence of one single imaginary vibration frequency and the normal vibrational mode. In addition, transition states were confirmed by the Intrinsic Reaction Coordinates (IRC) calculations. Unscaled zero point energies are included for species. All the gas-phase free energies (kJ/mol) reported in this paper correspond to the reference state of the intermediate **A**, at 373 K. The optimized structures were shown by Gauss View (Version 3.09) software to give high quality images of these structures.

ESI-MS of the intermediates

Figure S1. MS^2 using DMSO as the solvent

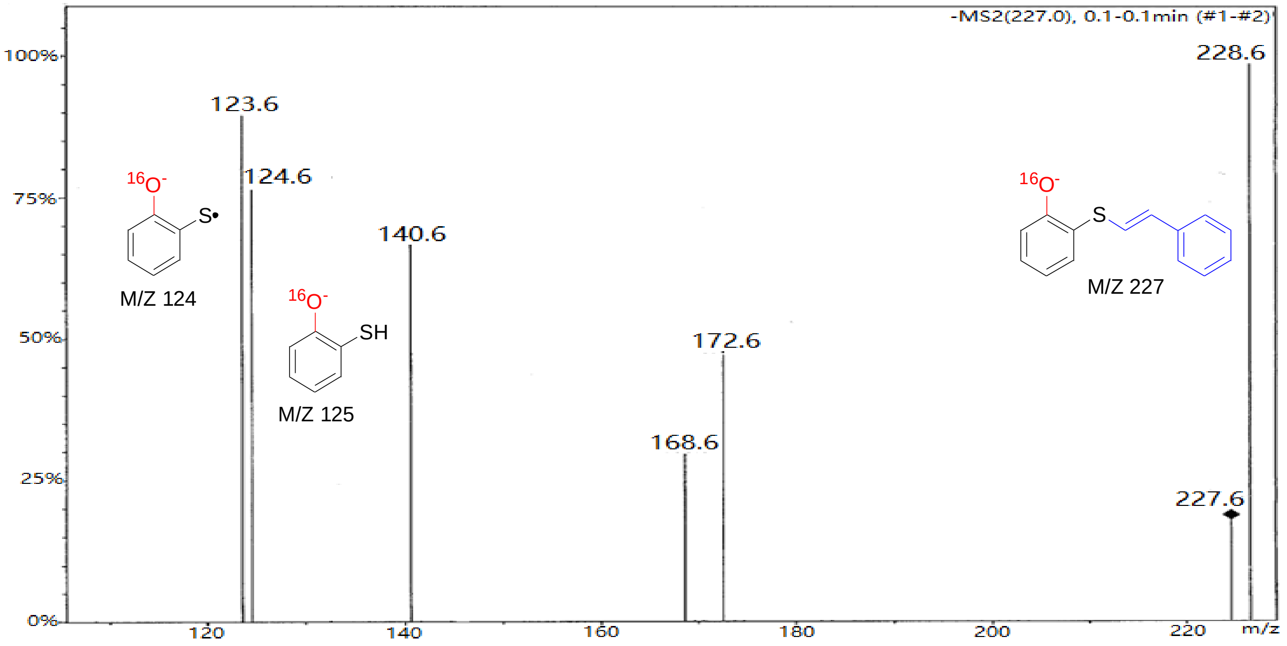

Figure S2. MS^2 using ^{18}O-DMSO as the solvent

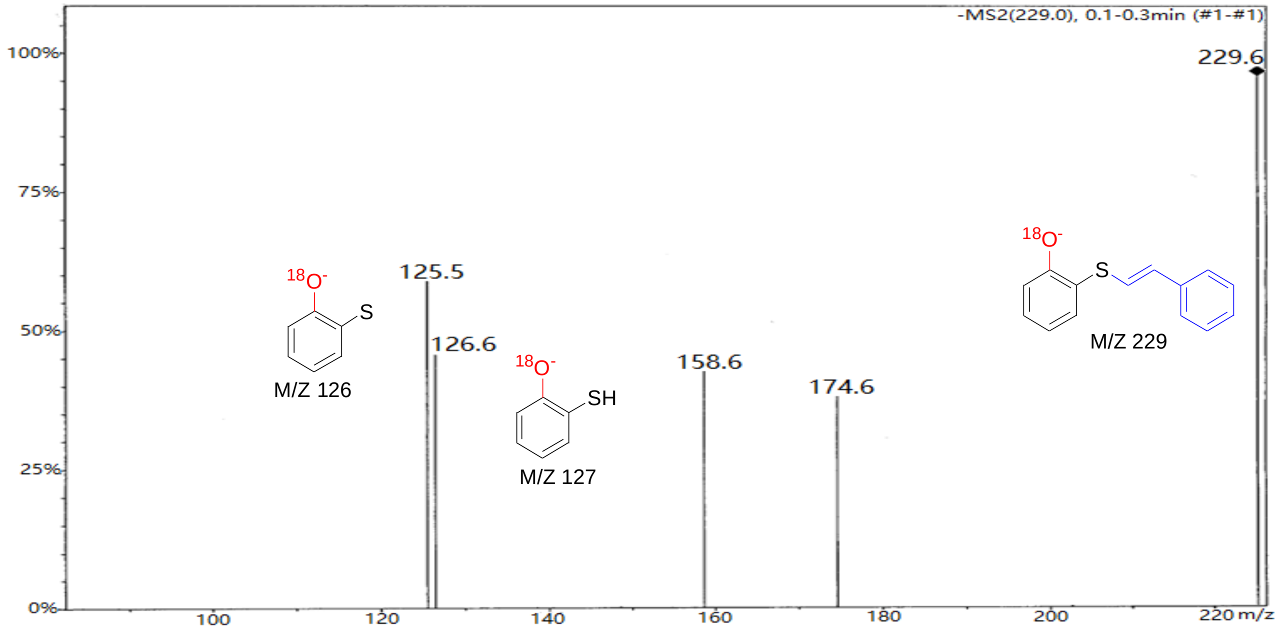

Figure S3. ESI-HR MS of intermediate A and A′

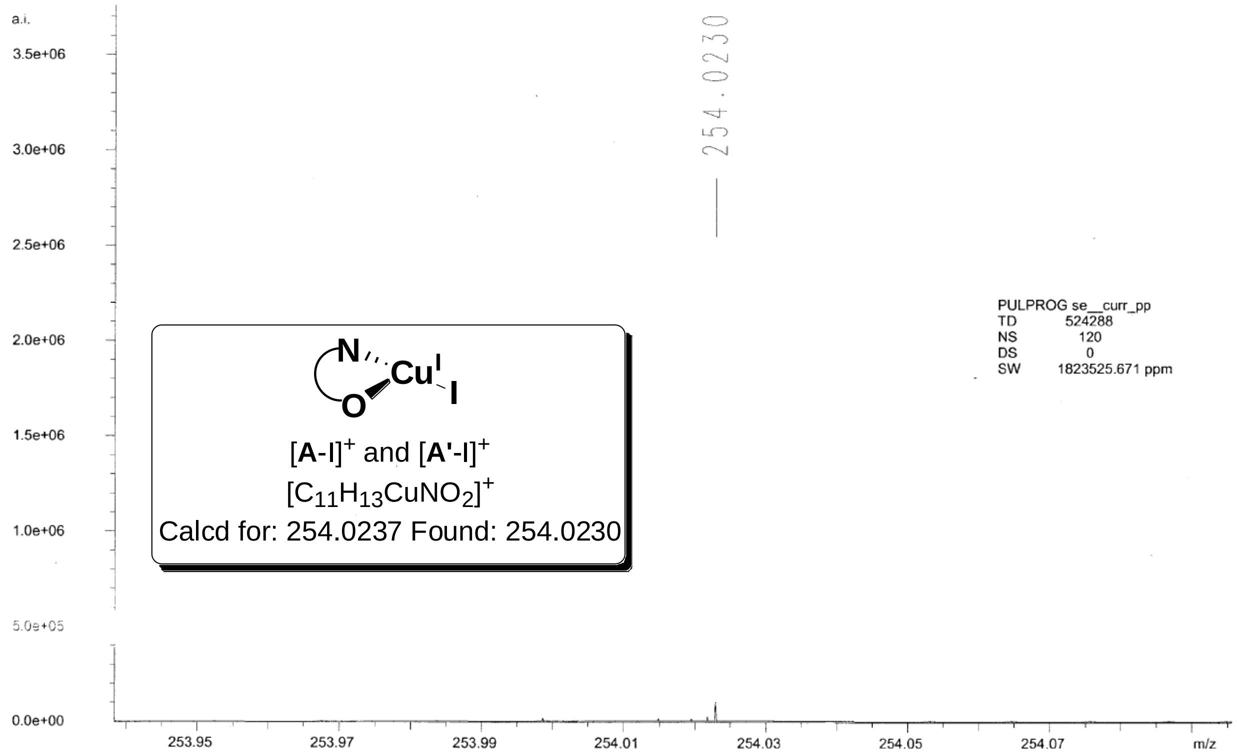

Figure S4. ESI-HR MS of intermediate B

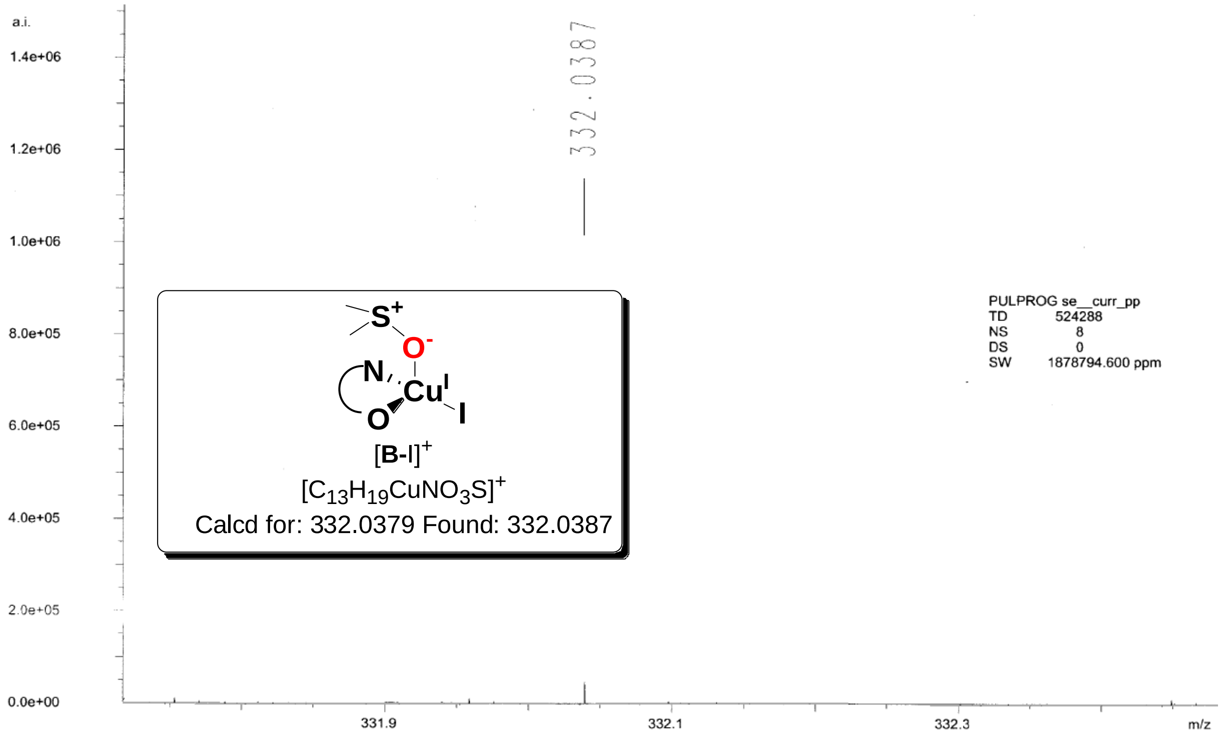

Figure S5. ESI-HR MS of intermediate C

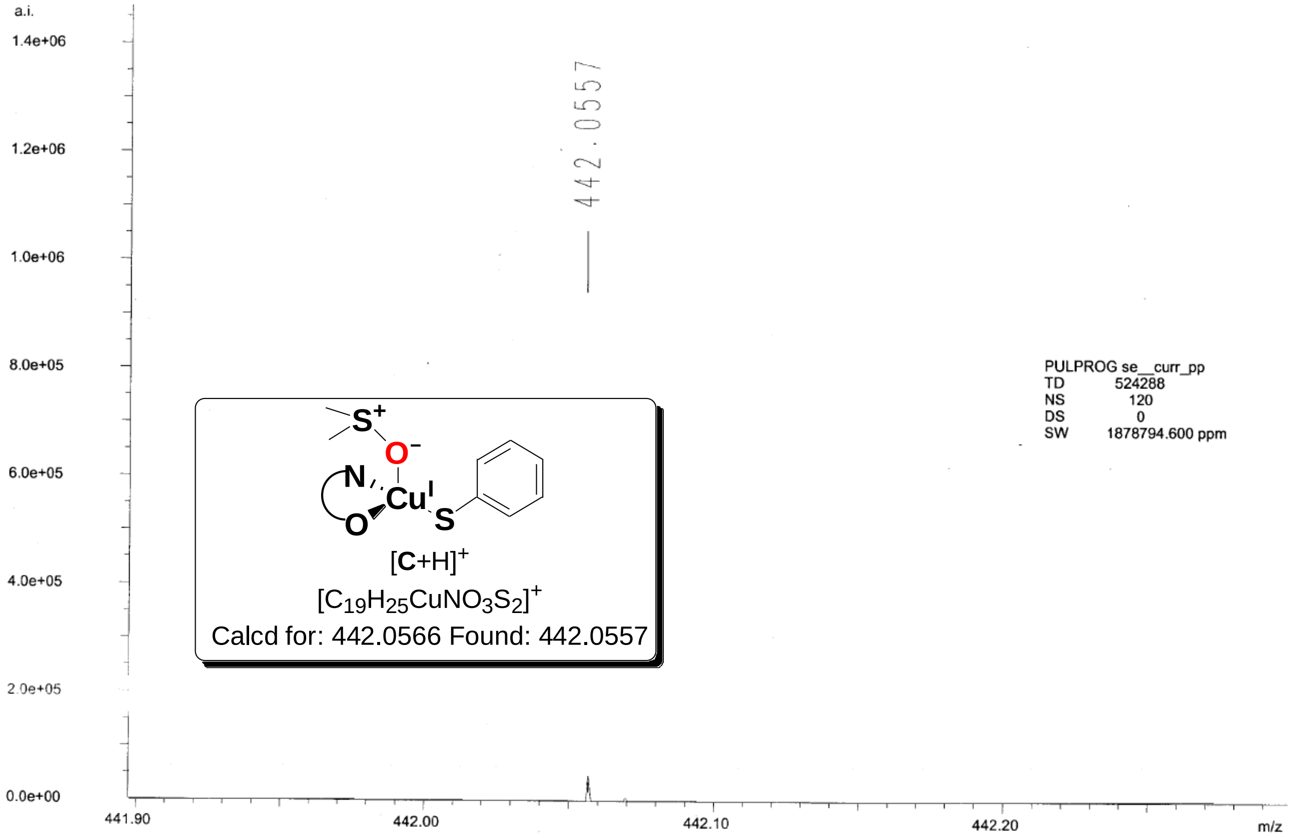

Figure S6. ESI-HR MS of intermediate D

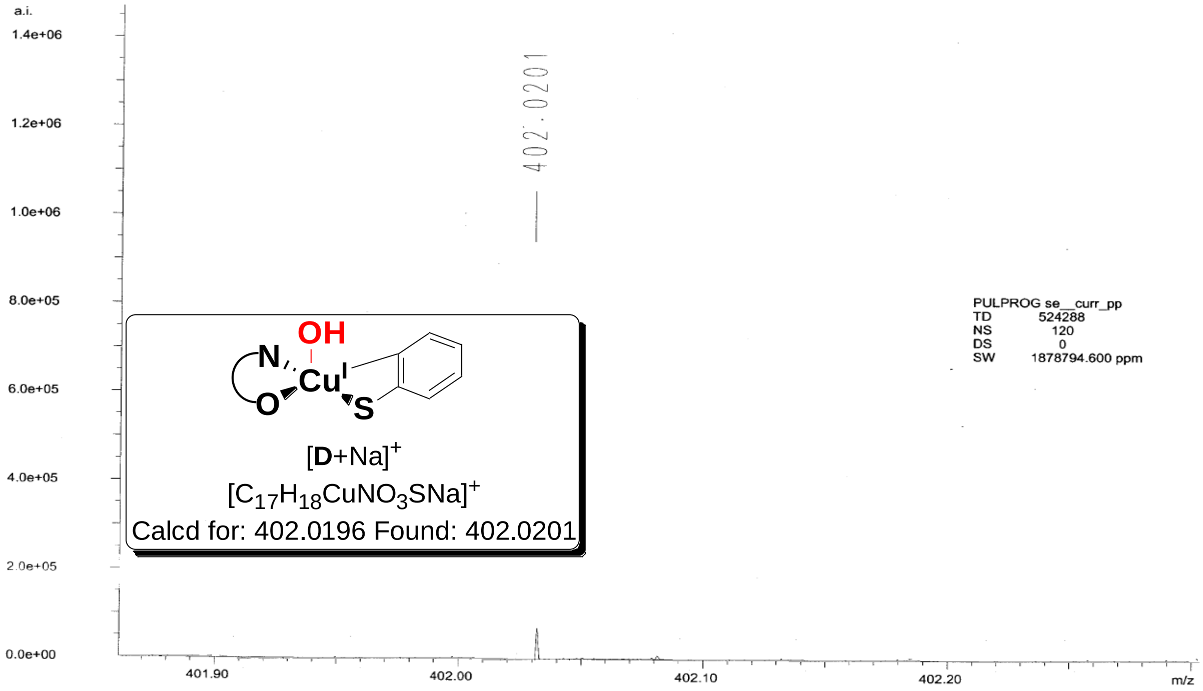

Figure S7. ESI-HR MS of intermediate E

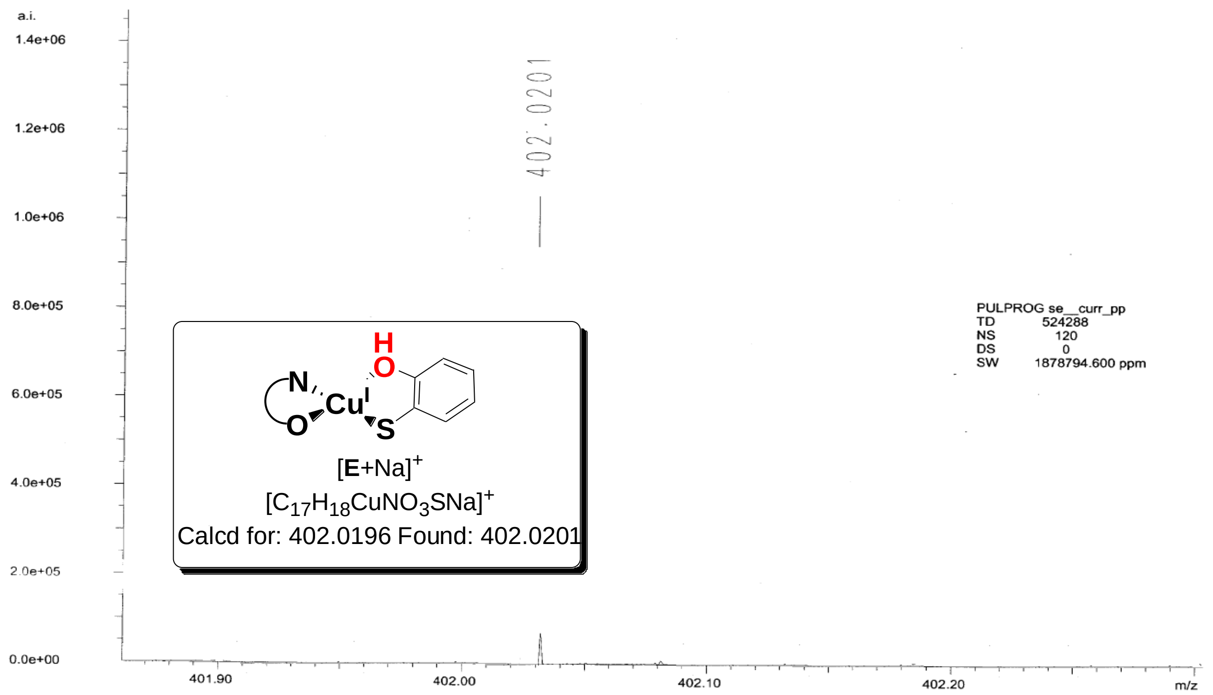

Figure S8. ESI-HR MS of intermediate F

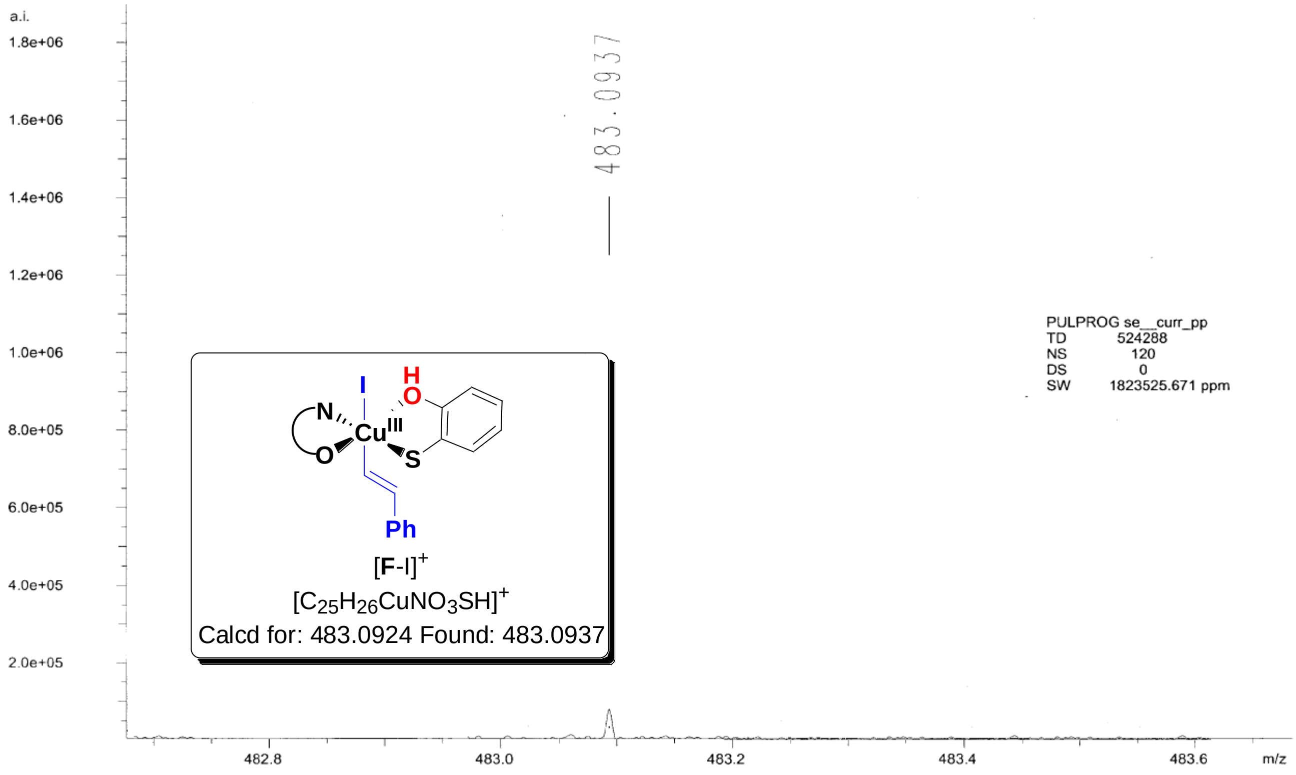

Figure S9. Geometries, total energies and zero point energy corrections

A	A′
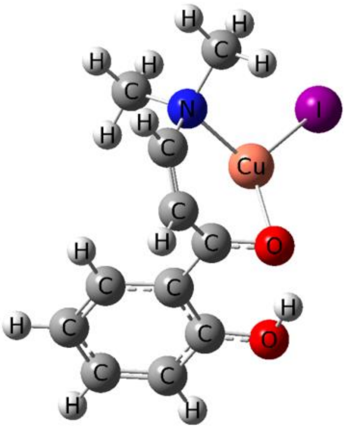	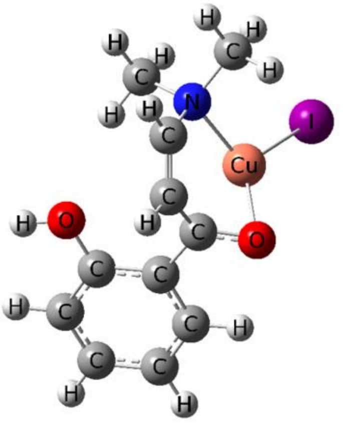
**B**	**C**
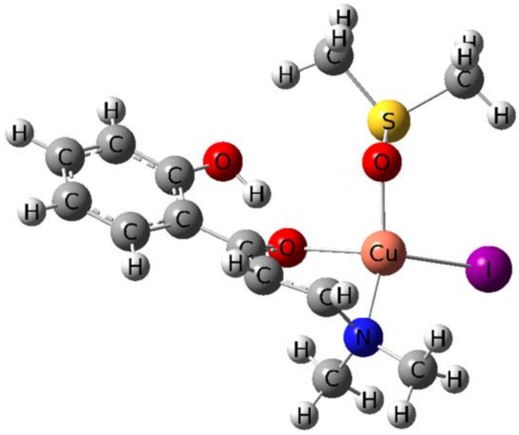	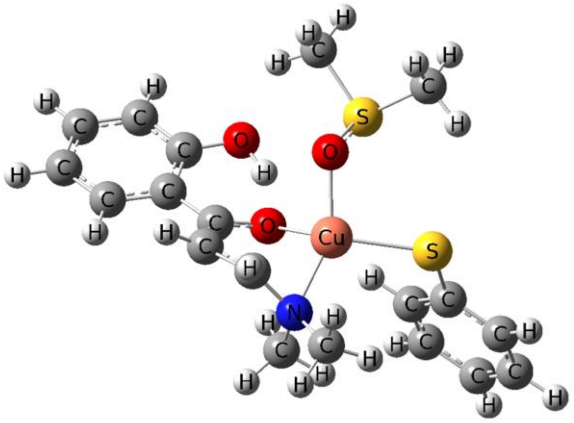
**C-TS**	**D**
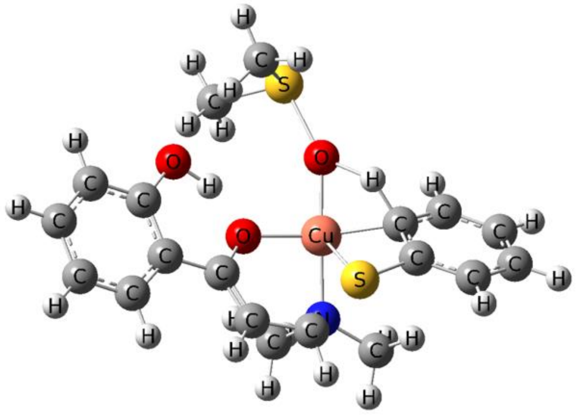	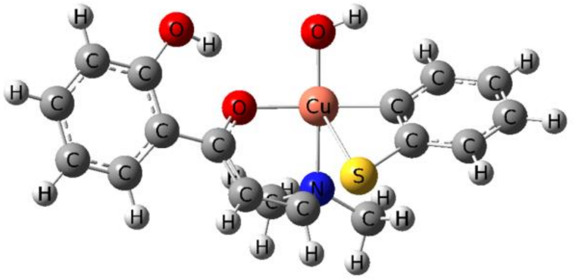
**E**	**E-TS**

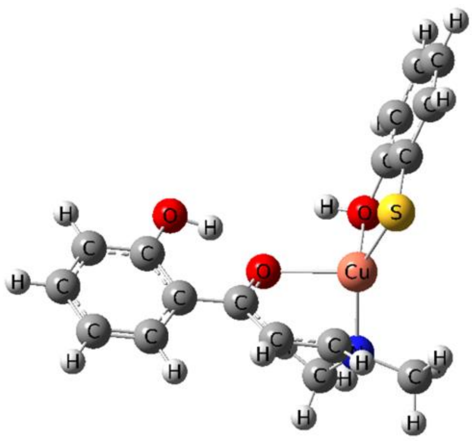	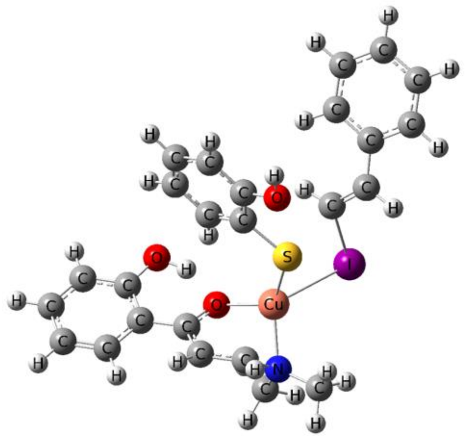
**F**	
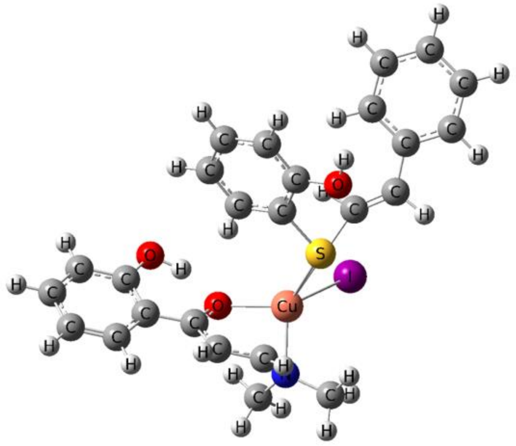	

A

----

opt freq b3lyp/gen Pseudo=Read test

Center Number	Atomic Number	Atomic Type	Coordinates (Angstroms) X	Y	Z
1	6	0	3.788739	-1.868588	0.323817
2	6	0	2.717842	-1.336853	-0.414484
3	6	0	2.366702	0.036065	-0.253797
4	6	0	3.114310	0.817444	0.657449
5	6	0	4.165850	0.282711	1.381317
6	6	0	4.498090	-1.071371	1.208162
7	1	0	4.033525	-2.915008	0.177021
8	1	0	2.853037	1.864234	0.781178
9	1	0	4.726395	0.902213	2.073420
10	1	0	5.320089	-1.502594	1.772337
11	6	0	1.255311	0.618699	-1.012747
12	8	0	0.585741	-0.125645	-1.852168
13	6	0	1.203238	2.120448	-1.153575
14	1	0	1.996806	2.613511	-1.711196
15	6	0	0.236126	2.827665	-0.572143
16	1	0	0.151360	3.912131	-0.651375
17	7	0	-0.773333	2.146732	0.225419
18	8	0	2.082421	-2.157858	-1.260743
19	1	0	1.368018	-1.639992	-1.700030
20	6	0	-0.525112	2.321479	1.679761
21	1	0	-0.590498	3.378864	1.970316
22	1	0	-1.272690	1.744206	2.228261
23	1	0	0.465996	1.937871	1.926343
24	6	0	-2.127927	2.643585	-0.125324
25	1	0	-2.869964	2.052152	0.413286
26	1	0	-2.235406	3.706129	0.132770
27	1	0	-2.292946	2.514779	-1.196728
28	29	0	-0.604606	0.256312	-0.399386
29	53	0	-2.532286	-1.266195	0.346818

Zero-point correction= 0.224553 (Hartree/Particle)
Thermal correction to Energy= 0.241108
Thermal correction to Enthalpy= 0.242052
Thermal correction to Gibbs Free Energy= 0.177732
Sum of electronic and zero-point Energies= -2283.701532

Sum of electronic and thermal Energies= -2283.684977
Sum of electronic and thermal Enthalpies= -2283.684033
Sum of electronic and thermal Free Energies= -2283.748353

A′

----
opt freq b3lyp/gen Pseudo=Read test

Center Number	Atomic Number	Atomic Type	Coordinates (Angstroms) X	Y	Z
1	6	0	-4.159901	-0.575731	-1.137036
2	6	0	-3.140780	0.212976	-0.593306
3	6	0	-2.318912	-0.290316	0.439185
4	6	0	-2.546990	-1.611652	0.871908
5	6	0	-3.556485	-2.397524	0.331757
6	6	0	-4.369110	-1.872939	-0.677308
7	1	0	-4.783048	-0.167899	-1.931235
8	1	0	-1.906351	-1.993675	1.658620
9	1	0	-3.711718	-3.409243	0.692835
10	1	0	-5.164216	-2.472319	-1.111755
11	6	0	-1.222539	0.471647	1.097525
12	8	0	-0.485358	-0.159322	1.947661
13	6	0	-1.276150	1.978207	1.145212
14	1	0	-2.122873	2.444358	1.645505
15	6	0	-0.328155	2.722990	0.580640
16	1	0	-0.310596	3.812874	0.629286
17	7	0	0.731064	2.087945	-0.188241
18	8	0	-2.914678	1.479551	-1.061362
19	1	0	-3.564092	1.674429	-1.756220
20	6	0	0.499079	2.248374	-1.646064
21	1	0	0.564195	3.306021	-1.941499
22	1	0	1.257826	1.672205	-2.182196
23	1	0	-0.492531	1.865425	-1.891418
24	6	0	2.052895	2.646576	0.183827
25	1	0	2.831730	2.093242	-0.344987
26	1	0	2.114243	3.714775	-0.071675
27	1	0	2.206441	2.524610	1.258670
28	29	0	0.646664	0.190167	0.463792
29	53	0	2.615337	-1.263259	-0.329333

Zero-point correction= 0.224344 (Hartree/Particle)
Thermal correction to Energy= 0.241241
Thermal correction to Enthalpy= 0.242185
Thermal correction to Gibbs Free Energy= 0.176977
Sum of electronic and zero-point Energies= -2283.665698
Sum of electronic and thermal Energies= -2283.648801
Sum of electronic and thermal Enthalpies= -2283.647856
Sum of electronic and thermal Free Energies= -2283.713065

B

----
opt freq b3lyp/gen Pseudo=Read test

Center Number	Atomic Number	Atomic Type	Coordinates (Angstroms) X	Y	Z
1	6	0	-4.922935	1.063230	-1.062145
2	6	0	-3.596710	0.631378	-0.915024
3	6	0	-3.310973	-0.550130	-0.180129
4	6	0	-4.390162	-1.280591	0.351348
5	6	0	-5.703449	-0.858745	0.190603
6	6	0	-5.964105	0.324721	-0.513681
7	1	0	-5.105153	1.973641	-1.624027
8	1	0	-4.185424	-2.207107	0.879074
9	1	0	-6.519462	-1.444054	0.602389
10	1	0	-6.987820	0.664534	-0.644422
11	6	0	-1.904852	-0.954473	0.025272
12	8	0	-0.994512	-0.433604	-0.732847
13	6	0	-1.530582	-1.818579	1.073929
14	1	0	-2.230216	-1.995961	1.881531
15	6	0	-0.171505	-2.074983	1.353935
16	1	0	0.100638	-2.326566	2.376365
17	7	0	0.836213	-2.418945	0.432914
18	8	0	-2.614468	1.378412	-1.467338
19	1	0	-1.791354	0.817105	-1.390509
20	6	0	2.039614	-3.058677	1.007740
21	1	0	1.885534	-4.141217	1.087376
22	1	0	2.893371	-2.839396	0.365488
23	1	0	2.241673	-2.643111	1.995471
24	6	0	0.480565	-2.984081	-0.886537
25	1	0	-0.225183	-2.330094	-1.391206

26	1	0	1.392972	-3.041275	-1.481663
27	1	0	0.052106	-3.983125	-0.748485
28	29	0	0.898750	-0.509560	0.483898
29	8	0	0.408533	1.134831	1.357065
30	16	0	0.463567	2.397578	0.436080
31	6	0	1.769971	3.424739	1.166217
32	1	0	1.596401	3.521311	2.240267
33	1	0	2.706109	2.899809	0.967088
34	1	0	1.771506	4.400210	0.672947
35	6	0	-0.992818	3.346899	0.957574
36	1	0	-1.048387	3.351693	2.048239
37	1	0	-0.911828	4.361768	0.559701
38	1	0	-1.858237	2.848224	0.517972
39	53	0	3.239239	0.142658	-0.785358

C

----

opt freq ub3lyp/6-31g(d)

Center Number	Atomic Number	Atomic Type	Coordinates (Angstroms) X	Y	Z
1	6	0	4.787960	-0.156760	2.003837
2	6	0	3.506375	-0.203405	1.438309
3	6	0	3.274782	-0.960950	0.260077
4	6	0	4.350967	-1.684413	-0.285258
5	6	0	5.617124	-1.649081	0.285433
6	6	0	5.832588	-0.872667	1.431283
7	1	0	4.930721	0.438496	2.900594
8	1	0	4.173610	-2.300347	-1.162198
9	1	0	6.429015	-2.223667	-0.150533
10	1	0	6.819033	-0.836788	1.886559
11	6	0	1.936812	-0.943935	-0.371066
12	8	0	0.920867	-0.564901	0.340591
13	6	0	1.761788	-1.241666	-1.732654
14	1	0	2.625536	-1.246397	-2.387513
15	6	0	0.498557	-1.080190	-2.351577
16	1	0	0.472479	-0.831421	-3.410575
17	7	0	-0.735410	-1.555078	-1.881451
18	8	0	2.520179	0.510644	2.033504

19	1	0	1.677852	0.205871	1.591077
20	6	0	-1.818908	-1.655316	-2.880912
21	1	0	-1.762558	-2.622657	-3.395705
22	1	0	-2.777794	-1.546178	-2.372660
23	1	0	-1.726140	-0.845988	-3.606708
24	6	0	-0.777676	-2.660803	-0.901571
25	1	0	-0.133834	-2.432426	-0.056770
26	1	0	-1.804095	-2.759209	-0.544526
27	1	0	-0.453953	-3.586457	-1.392301
28	29	0	-0.615760	0.215195	-1.128777
29	8	0	0.302297	1.932420	-1.173244
30	16	0	0.150450	2.718435	0.168821
31	6	0	-0.876710	4.157567	-0.253064
32	1	0	-0.475961	4.643081	-1.147017
33	1	0	-1.877581	3.760554	-0.437846
34	1	0	-0.893537	4.845337	0.597844
35	6	0	1.761192	3.544457	0.336710
36	1	0	2.047795	3.979630	-0.624070
37	1	0	1.687336	4.311886	1.112878
38	1	0	2.472068	2.776212	0.647987
39	16	0	-2.725594	0.929956	-0.531118
40	6	0	-3.426998	-0.127641	0.721592
41	6	0	-4.825802	-0.261076	0.801616
42	6	0	-2.644098	-0.823246	1.662095
43	6	0	-5.416307	-1.060434	1.779653
44	1	0	-5.446354	0.270390	0.085404
45	6	0	-3.239702	-1.627025	2.635508
46	1	0	-1.562060	-0.726935	1.632024
47	6	0	-4.628717	-1.752396	2.702827
48	1	0	-6.500258	-1.144673	1.817786
49	1	0	-2.610112	-2.152312	3.350540
50	1	0	-5.090092	-2.376799	3.463328

Zero-point correction= 0.398657 (Hartree/Particle)
Thermal correction to Energy= 0.427322
Thermal correction to Enthalpy= 0.428266
Thermal correction to Gibbs Free Energy= 0.336313
Sum of electronic and zero-point Energies= -3455.172462
Sum of electronic and thermal Energies= -3455.143796
Sum of electronic and thermal Enthalpies= -3455.142852
Sum of electronic and thermal Free Energies= -3455.234805

C-TS

----

opt=(calcfc,ts,noeigen) freq ub3lyp/6-31g(d) iop(1/8=5)

Center	Atomic	Atomic	Coordinates (Angstroms)		
Number	Number	Type	X	Y	
1	6	0	5.006172	1.391065	-0.446349
2	6	0	3.647462	1.153068	-0.183986
3	6	0	3.170199	-0.182763	-0.119219
4	6	0	4.094531	-1.228980	-0.289090
5	6	0	5.439615	-0.987135	-0.538379
6	6	0	5.890752	0.335625	-0.622993
7	1	0	5.336149	2.423838	-0.501787
8	1	0	3.742470	-2.252505	-0.196786
9	1	0	6.131115	-1.816171	-0.656781
10	1	0	6.940005	0.542870	-0.817659
11	6	0	1.729599	-0.455511	0.101059
12	8	0	1.086445	0.478948	0.798664
13	6	0	1.117916	-1.597616	-0.351563
14	1	0	1.667286	-2.285424	-0.981608
15	6	0	-0.324264	-1.853810	-0.219121
16	1	0	-0.564381	-2.912419	-0.351281
17	7	0	-0.946279	-1.367341	1.046922
18	8	0	2.856741	2.236769	-0.031948
19	1	0	1.965899	1.923378	0.248307
20	6	0	-2.269146	-2.022069	1.270433
21	1	0	-2.097666	-3.062355	1.573571
22	1	0	-2.803836	-1.494629	2.062128
23	1	0	-2.873725	-2.009451	0.369301
24	6	0	-0.127019	-1.685952	2.259454
25	1	0	0.808397	-1.138651	2.251181
26	1	0	-0.711536	-1.387266	3.134267
27	1	0	0.061579	-2.764180	2.308360
28	29	0	-0.808505	0.599838	0.656048
29	8	0	-0.733065	2.372670	0.376401
30	16	0	-1.265349	-0.982953	-1.737993
31	6	0	-2.704628	-0.140925	-1.061745
32	6	0	-3.952874	-0.247339	-1.695658
33	6	0	-2.585727	0.660417	0.079179
34	6	0	-5.045241	0.460833	-1.194380
35	1	0	-4.054692	-0.871315	-2.579215
36	6	0	-3.667838	1.367027	0.582073
37	6	0	-4.909316	1.257116	-0.054709

38	1	0	-6.007150	0.386834	-1.694279
39	1	0	-3.554952	2.011907	1.448407
40	1	0	-5.764324	1.801413	0.337175
41	1	0	-0.646574	2.762047	1.265780

Frequencies --	-354.7577	33.4659	42.1950
Red. masses --	1.0661	6.3783	6.0564
Frc consts --	0.0790	0.0042	0.0064
IR Inten --	100.5831	0.5155	0.1776

Zero-point correction= 0.320269 (Hartree/Particle)
Thermal correction to Energy= 0.341438
Thermal correction to Enthalpy= 0.342382
Thermal correction to Gibbs Free Energy= 0.271041
Sum of electronic and zero-point Energies= -2977.198386
Sum of electronic and thermal Energies= -2977.177217
Sum of electronic and thermal Enthalpies= -2977.176272
Sum of electronic and thermal Free Energies= -2977.247613

D

----
opt=calcfc freq ub3lyp/6-31g(d) iop(1/8=5)

Center Number	Atomic Number	Atomic Type	Coordinates (Angstroms) X	Y	Z
1	6	0	5.002473	1.395441	-0.463719
2	6	0	3.649091	1.158583	-0.170649
3	6	0	3.165762	-0.176616	-0.126314
4	6	0	4.082178	-1.222460	-0.340842
5	6	0	5.421420	-0.981551	-0.618177
6	6	0	5.876856	0.341003	-0.686593
7	1	0	5.335958	2.427856	-0.502166
8	1	0	3.729532	-2.246712	-0.261358
9	1	0	6.105600	-1.811035	-0.770992
10	1	0	6.921892	0.547850	-0.903280
11	6	0	1.728411	-0.449854	0.115193
12	8	0	1.091204	0.486096	0.811911
13	6	0	1.114595	-1.600759	-0.315709
14	1	0	1.657755	-2.293833	-0.945186
15	6	0	-0.322130	-1.865014	-0.164928

16	1	0	-0.553488	-2.929467	-0.261845
17	7	0	-0.942383	-1.346393	1.090209
18	8	0	2.873097	2.243260	0.030903
19	1	0	1.990761	1.932288	0.341296
20	6	0	-2.270383	-1.985536	1.329347
21	1	0	-2.108164	-3.025815	1.637175
22	1	0	-2.790402	-1.447974	2.124060
23	1	0	-2.883354	-1.971926	0.433906
24	6	0	-0.120821	-1.654658	2.305635
25	1	0	0.822696	-1.122211	2.281144
26	1	0	-0.695388	-1.330689	3.177661
27	1	0	0.050863	-2.734815	2.371373
28	29	0	-0.804092	0.603854	0.682030
29	8	0	-0.672868	2.356523	0.461355
30	16	0	-1.293021	-1.081429	-1.722800
31	6	0	-2.698242	-0.167109	-1.080182
32	6	0	-3.946841	-0.257788	-1.716160
33	6	0	-2.562437	0.665046	0.039524
34	6	0	-5.025504	0.489962	-1.241114
35	1	0	-4.062693	-0.904943	-2.581045
36	6	0	-3.639730	1.397449	0.524579
37	6	0	-4.880662	1.307033	-0.118545
38	1	0	-5.985563	0.424048	-1.745740
39	1	0	-3.521614	2.041751	1.392263
40	1	0	-5.726743	1.874236	0.259753
41	1	0	-1.459643	2.741183	0.046001

Zero-point correction= 0.321681 (Hartree/Particle)
Thermal correction to Energy= 0.343106
Thermal correction to Enthalpy= 0.344050
Thermal correction to Gibbs Free Energy= 0.272336
Sum of electronic and zero-point Energies= -2977.208926
Sum of electronic and thermal Energies= -2977.187501
Sum of electronic and thermal Enthalpies= -2977.186557
Sum of electronic and thermal Free Energies= -2977.258271

E

----

opt=calcfc freq ub3lyp/6-31g(d)

Center Atomic Atomic Coordinates (Angstroms)

Number	Number	Type	X	Y	Z
1	6	0	4.378513	-2.573112	-0.331735
2	6	0	3.218529	-1.790594	-0.453452
3	6	0	3.168154	-0.495911	0.135259
4	6	0	4.323030	-0.026242	0.796015
5	6	0	5.468982	-0.798047	0.903972
6	6	0	5.487746	-2.082996	0.340419
7	1	0	4.375467	-3.559601	-0.784460
8	1	0	4.317797	0.976026	1.212392
9	1	0	6.343822	-0.408819	1.415864
10	1	0	6.380092	-2.698400	0.421226
11	6	0	1.938508	0.312511	0.052305
12	8	0	0.974816	-0.079528	-0.721795
13	6	0	1.766828	1.492385	0.814370
14	1	0	2.359449	1.642548	1.708463
15	6	0	0.610330	2.290121	0.679684
16	1	0	0.368690	2.973292	1.490830
17	7	0	0.029119	2.678460	-0.562834
18	8	0	2.183827	-2.312681	-1.137150
19	1	0	1.490899	-1.600650	-1.151488
20	6	0	-0.876237	3.840309	-0.474831
21	1	0	-0.306158	4.779211	-0.465559
22	1	0	-1.550215	3.838646	-1.334811
23	1	0	-1.477652	3.767097	0.432956
24	6	0	0.863101	2.692231	-1.778349
25	1	0	1.218616	1.687486	-1.997488
26	1	0	0.246198	3.039893	-2.610402
27	1	0	1.715766	3.369389	-1.644712
28	29	0	-0.829652	1.032614	0.105152
29	8	0	-1.900076	-0.108774	-1.240541
30	16	0	-2.070978	0.298602	1.764002
31	6	0	-3.082000	-0.763878	0.761621
32	6	0	-4.113885	-1.529939	1.334550
33	6	0	-2.907838	-0.891374	-0.625496
34	6	0	-4.911524	-2.370192	0.562010
35	1	0	-4.274621	-1.459547	2.406387
36	6	0	-3.701824	-1.715445	-1.414594
37	6	0	-4.710064	-2.471672	-0.817293
38	1	0	-5.695614	-2.951193	1.040404
39	1	0	-3.529152	-1.756910	-2.487212
40	1	0	-5.331500	-3.124144	-1.423316
41	1	0	-1.250799	-0.688047	-1.673536

Zero-point correction= 0.320969 (Hartree/Particle)
Thermal correction to Energy= 0.343525
Thermal correction to Enthalpy= 0.344469
Thermal correction to Gibbs Free Energy= 0.267377
Sum of electronic and zero-point Energies= -2977.262567
Sum of electronic and thermal Energies= -2977.240012
Sum of electronic and thermal Enthalpies= -2977.239068
Sum of electronic and thermal Free Energies= -2977.316160

E-TS

----

opt=(calcfc,ts,noeigen) freq rb3lyp/6-31g(d) iop(1/8=5)

Center Number	Atomic Number	Atomic Type	Coordinates (Angstroms) X	Y	Z
1	6	0	-5.481638	2.144125	-1.755303
2	6	0	-4.432064	1.275201	-1.415809
3	6	0	-4.489125	0.537949	-0.200072
4	6	0	-5.634256	0.679925	0.611042
5	6	0	-6.672238	1.530249	0.264663
6	6	0	-6.585182	2.270133	-0.924473
7	1	0	-5.402230	2.698426	-2.685146
8	1	0	-5.706562	0.090337	1.520008
9	1	0	-7.544838	1.618001	0.904708
10	1	0	-7.392464	2.941331	-1.206019
11	6	0	-3.368001	-0.324993	0.196288
12	8	0	-2.441979	-0.589361	-0.674917
13	6	0	-3.216880	-0.830524	1.506463
14	1	0	-3.705830	-0.335420	2.337050
15	6	0	-2.143570	-1.690997	1.811634
16	1	0	-1.787946	-1.726743	2.838784
17	7	0	-1.739094	-2.805124	1.049932
18	8	0	-3.395302	1.187176	-2.271383
19	1	0	-2.807560	0.467252	-1.920897
20	6	0	-0.887259	-3.776076	1.763803
21	1	0	-1.499061	-4.451555	2.376317
22	1	0	-0.318561	-4.356737	1.035021
23	1	0	-0.178096	-3.242500	2.399380
24	6	0	-2.691523	-3.438143	0.118149
25	1	0	-2.979553	-2.733752	-0.659728

26	1	0	-2.192908	-4.289942	-0.348735
27	1	0	-3.579429	-3.780112	0.663972
28	29	0	-0.697566	-1.229905	0.389562
29	16	0	0.769511	0.157929	1.367817
30	6	0	0.468284	1.842209	0.908100
31	6	0	1.203883	2.871238	1.538216
32	6	0	-0.444968	2.198914	-0.096848
33	6	0	1.007701	4.204366	1.163223
34	6	0	-0.641837	3.528064	-0.466829
35	6	0	0.088996	4.533447	0.167075
36	1	0	1.580630	4.985909	1.660311
37	1	0	-1.361986	3.766457	-1.243613
38	1	0	-0.050476	5.574911	-0.109355
39	1	0	-1.015311	1.415548	-0.583191
40	6	0	2.412136	-0.275236	-0.265023
41	1	0	2.136120	0.554827	-0.902430
42	6	0	3.646810	-0.652293	0.094265
43	1	0	3.772016	-1.561484	0.678062
44	6	0	4.877545	0.093556	-0.194774
45	6	0	6.110841	-0.461015	0.201464
46	6	0	4.905765	1.340417	-0.852916
47	6	0	7.315108	0.191352	-0.053942
48	1	0	6.116521	-1.420756	0.713323
49	6	0	6.109865	1.988600	-1.109778
50	1	0	3.976245	1.811690	-1.160219
51	6	0	7.324850	1.421100	-0.713909
52	1	0	8.249526	-0.265142	0.263681
53	1	0	6.099892	2.948426	-1.620863
54	1	0	8.262508	1.932128	-0.914638
55	8	0	2.095279	2.518500	2.507450
56	1	0	2.551584	3.316191	2.817640
57	53	0	1.171797	-1.987188	-1.087599

Frequencies --	-333.6437	5.9236	15.4060
Red. masses --	7.3774	6.6693	6.5682
Frc consts --	0.4839	0.0001	0.0009
IR Inten --	464.4164	0.1576	0.1940

Zero-point correction= 0.444503 (Hartree/Particle)
Thermal correction to Energy= 0.477025
Thermal correction to Enthalpy= 0.477970
Thermal correction to Gibbs Free Energy= 0.373715
Sum of electronic and zero-point Energies= -3297.484351
Sum of electronic and thermal Energies= -3297.451829

Sum of electronic and thermal Enthalpies= -3297.450885
Sum of electronic and thermal Free Energies= -3297.555139

F

----
opt=calcfc freq b3lyp/6-31g(d)

Center Number	Atomic Number	Atomic Type	Coordinates (Angstroms) X	Y	Z
1	6	0	6.161135	1.309458	-0.143606
2	6	0	4.911420	0.687988	0.018847
3	6	0	4.341373	-0.046715	-1.059023
4	6	0	5.084224	-0.158168	-2.252787
5	6	0	6.320915	0.449232	-2.403202
6	6	0	6.853796	1.193077	-1.339190
7	1	0	6.562394	1.868886	0.695584
8	1	0	4.681117	-0.753979	-3.065949
9	1	0	6.872930	0.344107	-3.332309
10	1	0	7.822590	1.674618	-1.445448
11	6	0	3.012675	-0.663885	-0.914856
12	8	0	2.449489	-0.675035	0.250192
13	6	0	2.295855	-1.194502	-2.012344
14	1	0	2.520903	-0.854269	-3.016223
15	6	0	1.058209	-1.831835	-1.823869
16	1	0	0.345136	-1.825187	-2.646052
17	7	0	0.735497	-2.762566	-0.836811
18	8	0	4.301728	0.819542	1.209894
19	1	0	3.473378	0.271975	1.155859
20	6	0	-0.483255	-3.563035	-1.069531
21	1	0	-0.245160	-4.448639	-1.672666
22	1	0	-0.897010	-3.855820	-0.103406
23	1	0	-1.228189	-2.953403	-1.584023
24	6	0	1.800029	-3.527156	-0.158252
25	1	0	2.493312	-2.849523	0.334118
26	1	0	1.329888	-4.153597	0.600983
27	1	0	2.328245	-4.148826	-0.891493
28	29	0	0.299608	-1.033866	0.053000
29	16	0	-0.576817	0.954389	-0.722736
30	6	0	-0.176913	2.222134	0.486639
31	6	0	-0.612888	3.541299	0.265000

32	6	0	0.612664	1.933303	1.602113
33	6	0	-0.257996	4.545591	1.170801
34	6	0	0.973041	2.938959	2.498091
35	6	0	0.532472	4.243921	2.278917
36	1	0	-0.600206	5.564958	0.999999
37	1	0	1.590716	2.700013	3.357628
38	1	0	0.803159	5.036837	2.970592
39	1	0	0.935474	0.911012	1.760380
40	6	0	-2.309678	0.644369	-0.429923
41	1	0	-2.524779	0.188998	0.532651
42	6	0	-3.223539	0.892433	-1.380526
43	1	0	-2.891392	1.367900	-2.302333
44	6	0	-4.660518	0.599706	-1.302314
45	6	0	-5.502534	1.080042	-2.320559
46	6	0	-5.237377	-0.142843	-0.254114
47	6	0	-6.875687	0.842680	-2.290474
48	1	0	-5.070696	1.650295	-3.139966
49	6	0	-6.608192	-0.378906	-0.224577
50	1	0	-4.608742	-0.546513	0.534010
51	6	0	-7.434155	0.113251	-1.240354
52	1	0	-7.507905	1.225860	-3.087030
53	1	0	-7.035236	-0.954776	0.592027
54	1	0	-8.503795	-0.076210	-1.213967
55	8	0	-1.374921	3.791579	-0.838446
56	1	0	-1.595818	4.735893	-0.858455
57	53	0	-0.945645	-1.734752	2.104366

Zero-point correction= 0.447151 (Hartree/Particle)

Thermal correction to Energy= 0.479993

Thermal correction to Enthalpy= 0.480938

Thermal correction to Gibbs Free Energy= 0.376093

Sum of electronic and zero-point Energies= -3297.565213

Sum of electronic and thermal Energies= -3297.532371

Sum of electronic and thermal Enthalpies= -3297.531427

Sum of electronic and thermal Free Energies= -3297.636272

$C_6H_5S^-$

----

opt=calcfc freq rb3lyp/6-31g(d)

Center Atomic Atomic Coordinates (Angstroms)

Number	Number	Type	X	Y	Z
1	6	0	1.200031	-1.557390	0.000000
2	6	0	1.200948	-0.164884	0.000000
3	6	0	0.000000	0.598804	0.000000
4	6	0	-1.200948	-0.164884	0.000000
5	6	0	-1.200031	-1.557390	0.000000
6	6	0	0.000000	-2.279790	0.000000
7	1	0	2.152095	-2.090993	0.000000
8	1	0	2.144471	0.376436	0.000000
9	1	0	-2.144471	0.376436	0.000000
10	1	0	-2.152095	-2.090993	0.000000
11	1	0	0.000000	-3.368537	0.000000
12	16	0	0.000000	2.346928	0.000000

C_6H_5-CH=CH-I

----

opt freq b3lyp/gen Pseudo=Read test

Center Number	Atomic Number	Atomic Type	Coordinates (Angstroms) X	Y	Z
1	6	0	-4.159374	-1.095141	0.000979
2	6	0	-2.777136	-1.279343	0.000010
3	6	0	-1.898056	-0.182196	-0.000964
4	6	0	-2.451903	1.111835	-0.001301
5	6	0	-3.831009	1.295961	-0.000333
6	6	0	-4.692658	0.194012	0.000864
7	1	0	-4.818202	-1.959299	0.001782
8	1	0	-2.367611	-2.286758	0.000131
9	1	0	-1.801386	1.981524	-0.002658
10	1	0	-4.237445	2.303827	-0.000668
11	1	0	-5.768981	0.342020	0.001547
12	6	0	-0.449306	-0.447586	-0.001677
13	1	0	-0.175400	-1.500839	-0.005031
14	6	0	0.520669	0.471369	0.001741
15	1	0	0.362239	1.542245	0.005737
16	53	0	2.589423	-0.015777	0.000061

DMSO

----
opt freq rb3lyp/6-31g(d)

Center Number	Atomic Number	Atomic Type	Coordinates (Angstroms) X	Y	Z
1	16	0	0.000002	0.242862	-0.439836
2	8	0	0.000030	1.507929	0.387240
3	6	0	-1.363509	-0.821999	0.180197
4	1	0	-2.300117	-0.312834	-0.059660
5	1	0	-1.333401	-1.796545	-0.316877
6	1	0	-1.275341	-0.933018	1.265088
7	6	0	1.363481	-0.822045	0.180195
8	1	0	1.275322	-0.933027	1.265090
9	1	0	1.333325	-1.796603	-0.316852
10	1	0	2.300105	-0.312923	-0.059689

MeS

----
opt freq rb3lyp/6-31g(d)

Center Number	Atomic Number	Atomic Type	Coordinates (Angstroms) X	Y	Z
1	16	0	0.000000	-0.655426	-0.000001
2	6	0	-1.392709	0.507979	-0.000001
3	1	0	-2.306018	-0.093703	-0.000024
4	1	0	-1.400241	1.144587	0.892625
5	1	0	-1.400205	1.144642	-0.892586
6	6	0	1.392709	0.507980	-0.000001
7	1	0	1.400203	1.144649	-0.892582
8	1	0	1.400243	1.144580	0.892630
9	1	0	2.306019	-0.093702	-0.000032
